# Ethanol extract of *Scutellaria baicalensis *Georgi prevents oxidative damage and neuroinflammation and memorial impairments in artificial senescense mice

**DOI:** 10.1186/1423-0127-18-14

**Published:** 2011-02-08

**Authors:** Kukhuon Jeong, Yong-Cheol Shin, Sunju Park, Jeong-Su Park, Namil Kim, Jae-Young Um, Hoyeon Go, Seungho Sun, Sundong Lee, Wansu Park, Youkyung Choi, Yunkyung Song, Gyungjun Kim, Chanyong Jeon, Jonghyeong Park, Keysang Lee, Oksun Bang, Seong-Gyu Ko

**Affiliations:** 1 Center for Clinical Research and Genomics, Kyung Hee University, Seoul 130-701, Korea; 2 Institute of Oriental Medicine, Kyung Hee University, Seoul 130-701, Korea; 3 Semyung University, Chungju 380-080, Korea; 4 Sangji University, Wonju 220-702, Korea; 5 Kyungwon University, Seongnam 461-701, Korea; 6 Wonkwang University, Iksan 570-749, Korea; 7 Korean Institute of Oriental Medicine, Daejeon 305-811, Korea; 8 Cytokine Research Lab. Dept. of Experimental Therapeutics, University of Texas M. D. Anderson Cancer Center, Texas 77030, USA

## Abstract

Aging is a progressive process related to the accumulation of oxidative damage and neuroinflammation. We tried to find the anti-amnesic effect of the *Scutellaria baicalens *Georgia (SBG) ethanol extract and its major ingredients. The antioxidative effect of SBG on the mice model with memory impairment induced by chronic injection of D-galactose and sodium nitrate was studied. The Y-maze test was used to evaluate the learning and memory function of mice. The activities of superoxide dismutase, catalase and the content of malondialdehyde in brain tissue were used for the antioxidation activities. Neuropathological alteration and expression of bcl-2 protein were investigated in the hippocampus by immunohistochemical staining. ROS, neuroinflammation and apoptosis related molecules expression such as Cox-2, iNOS, procaspase-3, cleaved caspase-3, 8 and 9, bcl-2 and bax protein and the products of iNOS and Cox-2, NO, PGE2, were studied using LPS-activated Raw 264.7 cells and microglia BV2 cells. The cognition of mice was significantly improved by the treatment of baicalein and 50 and 100 mg/kg of SBG in Y-maze test. Both SBG groups showed strong antioxidation, antiinflammation effects with significantly decreased iNOS and Cox-2 expression, NO and PGE2 production, increased bcl-2 and decreased bax and cleaved caspase-3 protein expression in LPS induced Raw 264.7 and BV2 cells. We also found that apoptotic pathway was caused by the intrinsic mitochondrial pathway with the decreased cleaved caspase-9 and unchanged cleaved caspase-8 expression. These findings suggest that SBG, especially high dose, 100 mg/kg, improved the memory impairments significantly and showed antioxidation, antiinflammation and intrinsic caspase-mediated apoptosis effects.

## Background

Traditionally, *Scutellaria baicalensis *Georgi (SBG) has been widely used to treat high fever, jaundice and infection in the form of decoction or extracts. Several studies have reported that major compounds, such as baicalin and baicalein isolated from this medicinal herb showed antioxidative, antiinflammatory effects [1-5]. Those effects of baicalin and baicalein were could have originated from the traditional effects of the original herb of SBG. The brain is susceptible to free-radical damage due to its comparatively high levels of oxygen metabolism and also relatively deficient in both free-radical scavenging enzymes and antioxidant molecules as compared with other organs [6,7]. Oxidative stress by the imbalance between free radicals and the antioxidant system is a prominent and early feature in the pathogenesis of neuronal damage [8,9].

Until now, several models such as amyloid beta, aluminum-maltolate, senescence-accelerated, natural senescent model and D-galactose and sodium nitrate model have been used to mimic the pathophysiological alterations of senile dementia [10-13]. D-galactose can induce caspase-mediated apoptosis, inflammation and oxidative damage in the nervous system [14] and sodium nitrite (NaNO_2_) injection may cause ischemia and hypoxia in many organs in animals [15]. Thus, the model induced by D-galactose and NaNO_2 _is considered to be optimistic to induce the senescent syndromes, especially memory impairment with neuroinflammation and ischemia in animals similar to the aging patterns of human beings.

Inflammation is critical in recruiting immune cells and molecules to the site of infection for defense. Macrophage plays a central role in organizing the release of inflammation mediators, including prostaglandin E2 and nitric oxide as well as causing pathological consequences such as tissue edema and abnormal histological change [16,17].

We tried to find anti-amnesic effects of SBG and its major ingredients on the mice model with memory impairment induced by chronic injection of D-galactose and NaNO_2_. The major ingredients were investigated only in the Y-maze test, directly implicating the anti-amnesic effect since the high dose SBG group showed better effect than the baicalin and baicalein groups in this test. Because this animal memory deficit model was caused by oxidative damage and apoptosis by chronic injection of D-galactose and sodium nitrate, we evaluated the antioxidative effects of superoxide dismutase, catalase and malodialdehyde with brain tissues and checked photomicrographs of Cresyl violet-stained neuropathological changes and immunohistochemistry of mouse hippocampus cells incubated with bcl-2, typical anti-apoptotic molecule, monoclonal antibody in the hippocampus regions of senescent mice. Immortalized murine microglia cell lines, BV2, is widely used to study the neuroinflammatory mechanism in vitro, because this cell line retains most of the morphological and functional properties described for primary microglia [18]. Since we used the BV2 cell lines for this study and also used representative cell lines of Raw 264.7 cells for going into the particulars on the mechanism of antiinflammation and the protective effects of cell death by SBG, we confirmed antioxidative, anti-inflammatory and casepase dependent apoptotosis effects at the level of cell line of macrophage Raw 264.7 and microglia BV2. Prostaglandin E2 (PGE2) regulated by cyclooxygenase-2 (COX-2) and nitric oxide (NO) production induced by LPS through inducible nitric oxide synthase (iNOS) were investigated.

## Materials and methods

### Plants, compounds and chemical reagents

The SBG was purchased from Beijing Tongrentang (Beijing, China) and the ground powder was extracted twice with 80% (v/v) ethanol using an ultra-sonicator (Branson, USA.) and evaporated at 60°C and then freeze-dried. The final yield was 48.75 g (24.3%). The chromatogram of baicalein and baicalin were recorded at 315 nm and 272 nm respectively. HPLC (Shimadzu, Japan) analysis content of baicalin and baicalein was 4.1522% and 3.3075%, respectively in SBG. Baicalin and baicalein which were used for experiments were purchased from Waco (Osaka, Japan). D-Galactose, NaNO_2_, and LPS (Sigma-Aldrich, USA), Commercial kits for malondialdehyde, superoxide dismutase and catalase (Cayman, USA) were purchased. Dulbecco's modified Eagle's medium (DMEM), fetal bovine serum (FBS), penicillin and streptomycin were purchased from Gibco Life Technologies (MD, USA). COX-2, iNOS, bcl2, bax, procaspase-3, cleaved caspase-3, 8 and 9, and peroxidase-conjugated secondary antibody were purchased from Santa Cruz Biotechnology (CA, USA).

The enzyme immunoassay (EIA) kits used for the determination of nitric oxide, prostaglandin E2 were obtained from Assay Designs Inc. (MI, USA).

### Animals and administraion

ICR female mice (4 weeks old, 20-22 g) were purchased from Orient Bio Experimental Animal Center (Seongnam, Korea). Mice were housed (8 mice per cage) in a regulated environment at 25 ± 1°C with a 12 h/12 h light/dark cycle and with free access to standard rodent pellets (Purina, Korea). Animal care and experimental procedures followed requirements put forth in the Guide for the Care and Use of Laboratory Animals (Department of Health and Education, and Welfare, National Institute of Health, 1996). After 7 days of adaptation, the mice of 36 were randomly divided into six groups. The normal control group (NC, saline 0.3 ml, n = 6), aging control group (AC, D-galactose 120 mg/kg, NaNO_2 _90 mg/kg, n = 6), baicalin and baicalein treated group (200 mg/kg, n = 6 respectively), SBG treated group (50 mg/kg and 100 mg/kg, n = 6 respectively). Normal group mice were intraperitoneally injected saline 0.3 ml once daily for 60 days, and orally administered with saline 0.3 ml/mouse for 14 days from day 47 of the experiment. Mice in aging control and each treatment groups were intraperitoneally injected with D-galactose (120 mg/kg) and NaNO_2 _(90 mg/kg) once daily for 60 days. From day 47 to 60 for 2 weeks, aging control mice were orally administered with saline 0.3 ml/mouse, and the treatment groups of mice were treated with baicalin 200 mg/kg, baicalein 200 mg/kg, SBG 50 mg/kg and SBG 100 mg/kg for 14 days (orally, once daily).

### Cell culture

The murine macrophage Raw 264.7 cells and mouse microglia BV2 cells were obtained from the Korea Cell Line Bank (Seoul, Korea) and cultured in DMEM supplemented with 2 mM L-glutamine, 100 U/ml penicillin, 100 mg/ml streptomycin, and 10% heat-inactivated fetal bovine serum. The cells were subcultured twice weekly and grown on 6-well plates at 1 ×10^6 ^cells/well at 37 °C in fully humidified 5% CO_2 _air.

### MTT assay

The cell viability was assessed based on the content of metabolized blue formazan from 3-(4,5-dimethyl-thiazol-2-yl)-2,5-diphenyl tetrazolium bromide (MTT) converted by mitochondrial dehydrogenases in live cells.

### Y-maze test

For Y-maze test, the experimental mice were retained and subjected to training and test on day time to assess short-term and spatial memory performance in Y-maze. The maze was placed in a separate room with enough light. The Y-maze test was of 10 minutes duration and allowed the mice to explore only two arms (start arm and the other arm) of the maze, with the third arm (novel arm) blocked. Data was expressed as percentage of alternation calculated as (successive triplet sets/total number of arm entries-2) × 100.

### Brain tissue preparation

After examination of the memory behavior, all mice were deeply anesthetized and decapitated, and their brains were removed rapidly and homogenized in 50 mM (PH 7.4) cold phosphate buffer saline solution (PBS) containing a protease inhibitor cocktail (Sigma-Aldrich, USA) with 10 strokes at 1200 rpm in a homogenizer. Three brains of mice chosen randomly from each group were post-fixed in 4% paraformaldehyde in PBS (pH 7.4) overnight at 4°C, and then placed in a solution of 30% sucrose, 4% paraformaldehyde in PBS (pH 7.4).

### Crystal violet staining and immunohistochemisty

The sections were incubated with antibody of bcl2 (Santa Cruz, USA). The sections were General ABC Procedure. Fixed brains were cut into 30 um sections on a sliding microtome and the sections stained with Crystal violet. Slides were immersed for 5 min in each of the following: xylene, 100% alcohol, 95% alcohol, and 70% alcohol. They were dipped in distilled water and stained in 0.5% crystal violet for 15~30 min. They were differentiated in water for 3~5 min and then dehydrated through 70% alcohol, 95% alcohol, and 100% alcohol. They were then put in xylene and cover-slipped.

### Assay of SOD and CAT activities

The assay for total superoxide dismutases (SOD) is based on the ability to inhibit the oxidation of oxymine by the xanthine-xanthine oxidase system [19]. Brain homogenates was directly centrifuged at 8000 g for 10 minutes to obtain supernatants to assay brain catalase (CAT) level and the supernatant collected for determination of superoxide dismutase (SOD) activities. The hydroxylamine nitrite produced by the oxidation of oxymine had an absorbance peak at 550 nm. One unit (U) of SOD activity was defined as the amount that reduced the absorbance at 550 nm by 50%, and data were expressed as units per microgram of brain protein. Catalase activity was assayed by the previous method [20]. In brief, to a quartz cuvette, 0.65 ml of the phosphate buffer (50 mmol l^-1^; pH7.0) and 50 ul sample were added, and the reaction was started by addition of 0.3 ml of 30 mM hydrogen peroxide (H_2_O_2_). The decomposition of H_2_O_2 _was monitored at 240 nm at 25°C. CAT activity was calculated as nM H_2_O_2 _consumed in 1 min per milligram of brain protein.

### Measurement of MDA level

The level of lipid peroxidation in brain homogenate was indicated by the content of malondialdehyde (MDA) in brain tissue. The brain homogenates was sonicated four times for 30 seconds with 20 seconds intervals using a ultrasonicator (Branson, USA), centrifuged at 5000 g for 10 minutes at 4 °C. Thiobarbituric acid reaction (TBAR) method was used to determine the MDA which can be measured at the wave length of 532 nm by reacting with thiobarbituricacid (TBA) to form a stable chromophoricproduction. MDA content was expressed as nmol per milligram of brain protein. Protein concentration was measured using the method of Bradford [21]. Bovine serum albumin was used as standard.

### Nitrate assay

The nitrite which accumulated in culture medium was measured as an indicator of NO production according to the Griess reagent. The culture supernatant (100 ul) was mixed with 100 ul of Griess reagent [equal volumes of 1% (w/v) sulfanilamide in 5% (v/v) phosphoric acid and 0.1% (w/v) naphthyl ethylenediamine-HCl] for 10 minutes, and then the absorbance at 540 nm was measured in a microplate reader. Fresh culture medium was used as the blank in all experiments. The amount of nitrite in the samples was determined with reference to a sodium nitrite standard curve.

### PGE2 assay

The cells were incubated with the SBGs or LPS or both for 18 h. After incubating the cells for 18 h, the culture medium was collected and the concentration of PGE2 secreted into the culture media was measured using a specific enzyme immunoassay according to the manufacturer's instructions (Assay Design Inc., USA).

### Western blottings

RAW 264.7 cells and mouse microglia BV2 cells were collected by centrifugation and washed once with phosphate-buffered saline (PBS). The washed cell pellets were resuspended in extraction lysis buffer (50 mM HEPES pH 7.0, 250 mM NaCl, 5 mM EDTA, 0.1% Nonidet P-40, 1 mM phenylmethylsulfonyl fluoride, 0.5 mM dithiothreitol, 5 mM Na fluoride, and 0.5 mM Na orthovanadate) containing 5 ug/ml each of leupeptin and aprotinin and incubated with 20 min at 4 C. Cell debris was removed by microcentrifugation, followed by quick freezing of the supernatants. The protein concentration was determined using the Bio-Rad protein assay reagent according to the manufacturer's instructions. Forty micrograms of cellular protein from treated and untreated cell extracts was electroblotted onto a PVDF membrane following separation on a 10% SDS-polyacrylamide gel electrophoresis. The immunoblot was incubated overnight with blocking solution (5% skim milk) at 4°C, followed by incubation for 4 h with a primary antibody (Santa Cruz Biotechnology, USA). Blots were washed four times with Tween 20/Tris-buffered saline (TTBS) and incubated with a 1:1000 dilution of horseradish peroxidase-conjugated secondary antibody (Santa Cruz, USA.) for 1 h at room temperature. Blots were again washed three times with TTBS, and then developed by enhanced chemiluminescence (Amersham Life Science, USA).

### Statistical analysis

All data in the text are expressed as mean ± standard deviation (mean ± S.D.), and analyzed by one-way ANOVA and multiple comparisons were performed by Tukey HSD test. A criterion of *P *< 0.1 (* or #), 0.05 (** or ##) and 0.01 (*** or ###) were accepted as statistically significant and marked with * or #.

## Results

### Improvement of spontaneous alteration behaviors

**Figure 1:**
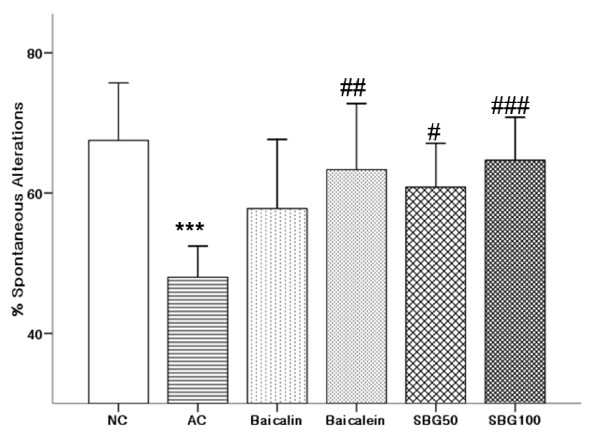
**The effects of ingredients and ethanol extraction of Scutellaria Radix on the changes of spontaneous alternation in the Y-maze test**. NC: normal control group, AC: aging control group, baicalin and baicalen treatment group (200 mg/kg), SBG50 and SBG100 group (*Scutellari baicalensis *Geroge treatment group, 50 mg/kg and 100 mg/kg). Values are expressed as mean ± standard deviation (mean ± S.D.), and analyzed by one-way ANOVA and multiple comparisons were performed by Tukey HSD test. A criterion of significance was accepted and marked as *P *< 0.1 (#), 0.01 (##) and 0.001 (*** or ###). The mark of star is AC group versus NC group and the mark of pound is each treatment group versus AC group.

In the Y-maze, the percentage of spontaneous alternation was calculated as an index of short-term and spatial memory. In the aging control (AC) group, the percentage of spontaneous alternation was significantly reduced as compared with the normal control (NC). The administration of baicalein (p = 0.046), SBG50 (p = 0.087), and SBG100 (p = 0.005) significantly improved the induced impairment of spontaneous alternation behavior as compared with the aging control (AC) but baicalin did not (p = 0.414) (Figure 1).

### Anti-oxidation activities

**Figure 2:**
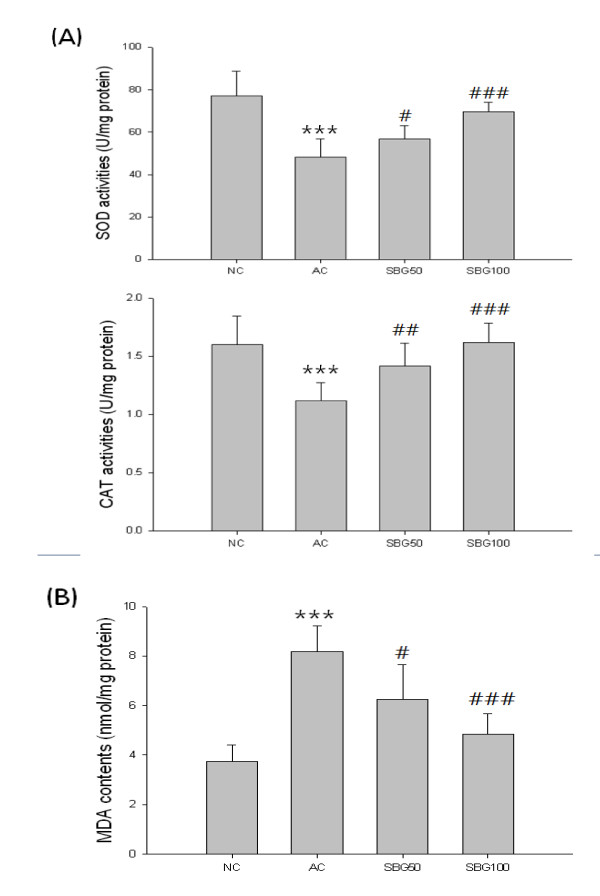
**The effect of Scutellaria Radix on SOD and catalase activities and MDA levels in the brain tissue**. (A) SOD and catalase activities, (B) MDA levels in hippocampus tissues. Values are expressed as mean ± standard deviation (mean ± S.D.), and analyzed by one-way ANOVA and multiple comparisons were performed by Tukey HSD test. A criterion of significance was accepted and marked as *P *< 0.1 (#), 0.01 (##) and 0.001 (*** or ###). The mark of star is AC group versus NC group and the mark of pound is each treatment group versus AC group.

SOD, catalase and MDA level in the brain tissues were determined as the biomarkers of oxidative stress in D-galactose and NaNO_2_-injected mice. The results show that chronic injection of D-galactose and NaNO_2 _induced significant decrease of SOD and catalase activities and increase of the MDA level. Additionally, we could find that only the high dose SBG 100 mg/kg treatment group showed strong antioxidative effects significantly in SOD, catalase activities (Figure 2A) and MDA levels (Figure 2B).

### Protection of neuropathological changes

**Figure 3:**
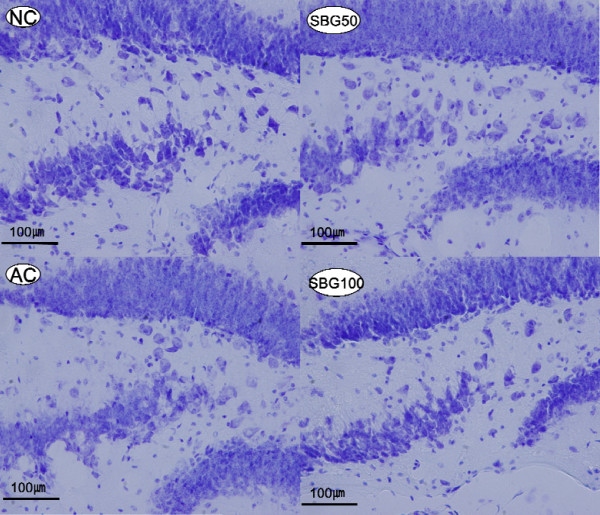
**Neuroprotective effects of SBG against D-galactose and NaNO_2 _induced senescent mice**. Representive photomicrographs of Cresyl violet-stained neuropathological changes in the hippocampus regions of senescent mice. In NC group, the neurons were full and arranged tightly, the nuclei are light-stained (NC). Cytoplasm of neurons of AC group was shrunken, the nuclei were side-moved and dark-stained, vacuoles were observed everywhere (AC). In the treatment group with 50 mg/kg or 100 mg/kg of SBG, the neurons were normalized, the nuclei were light-stained and arranged tightly and in the high dose group that is seen apparently (SBG50 and SBG100). Neuronal cell density in hippocampus region was measured by Nissl staining and cell counting.

Crystal violet staining showed that there were typical neuropathological changes in hippocampus of senescent mice induced by D-galactose and NaNO_2 _in AC group. In comparison with NC group, round, condensed, and dark stained neurons, neurofibrillary degeneration and vacuoles were observed in hippocampus of AC group mice. Consecutive administration of SBG (50 mg/kg, 100 mg/kg) remarkably attenuated these neuropathological changes. The neuronal cells recovered in their characteristic shapes with prolonged neurofibrillary (Figure 3).

### Inhibition of pro-inflammatory protein and related products

**Figure 4:**
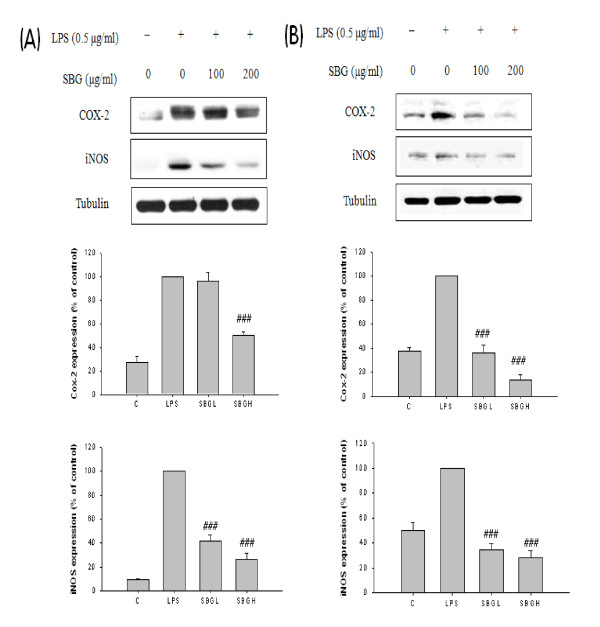
**The effect of SBG on LPS induced expression of COX-2 and iNOS protein with the Raw 264.7 cells and BV2 cells**. The Raw 264.7 cells (A) and BV2 cells (B) were pretreated with the indicated concentrations of SBG (SBG100, 100 ug/ml, SBG200, 200 ug/ml) for 30 min before incubation with LPS for 12 h. The cells were lysed and the lysates were analyzed by immunoblotting using anti COX-2, iNOS. Values are expressed as mean ± standard deviation (mean ± S.D.), and analyzed by one-way ANOVA and multiple comparisons were performed by Tukey HSD test. A criterion of significance was accepted and marked as *P *< 0.001 (###). The mark of star is AC group versus NC group and the mark of pound is each treatment group versus AC group.

**Figure 5:**
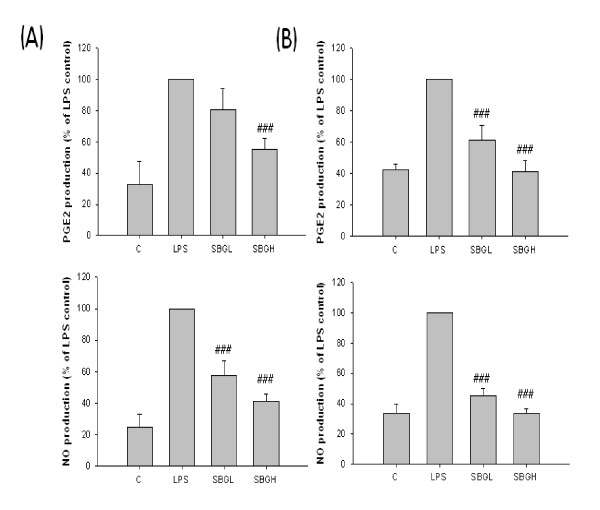
**The effect of SBG on LPS induced PGE2 and NO production with the Raw 264.7 cells and BV2 cells**. Raw 264.7 cells (A) and BV2 cells (B) were pretreated with the indicated concentrations of SBG for 30 min before incubation with LPS for 24 h. The culture supernatants were subsequently isolated and analyzed for nitrate levels. Values are expressed as mean ± standard deviation (mean ± S.D.), and analyzed by one-way ANOVA and multiple comparisons were performed by Tukey HSD test. A criterion of significance was accepted and marked as *P *< 0.001 (###). The mark of star is AC group versus NC group and the mark of pound is each treatment group versus AC group.

To investigate whether SBG can inhibit LPS-induced Cox-2 and iNOS expression, Raw 264.7 and microglia BV2 cells were pretreated for 30 min with 100 ug/ml and 200 ug/ml concentrations of SBG and subsequently treated with 0.5 ug/ml LPS. Raw 264.7 cells pretreated with SBG showed a dose-dependent inhibition of iNOS protein expression following LPS stimulation, but Cox-2 was only inhibited in high dose of SBG treatment group in Raw 264.7 cells (Figure 4A). But microglia BV2 cells significantly inhibited both Cox-2 and iNOS expression in both low and high doses of SBG (Figure 4B). Macrophage-derived NO and PGE2 are an important host defense and microbial and tumor cell killing agent, as well as a regulator of proinflammatory genes in vivo [22]. High dose of SBG group in Raw 264.7 and both dose of SBG groups in BV2 cells showed a dose-dependent inhibition of NO and PGE2 production following LPS stimulation (Figure 5A and 5B).

### Regulation of apoptotic proteins and immunohistochemal confirmation of bcl-2 protein

**Figure 6:**
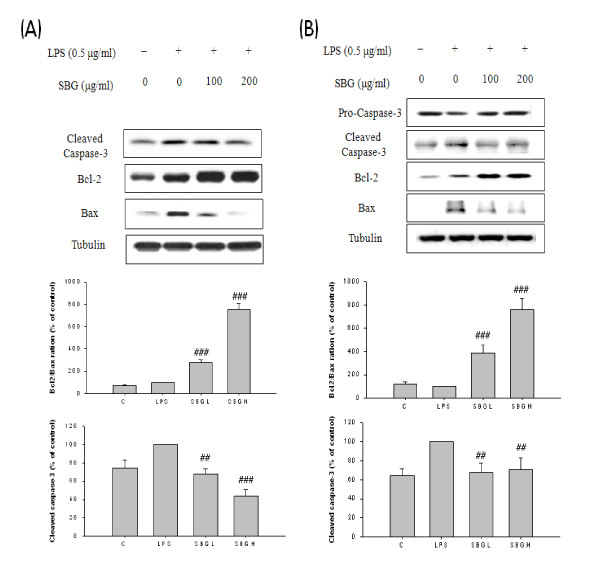
**The effect of SBG on the caspase-dependent apoptosis molecules in Raw 264.7 and BV2 cells**. The Raw 264.7 (A) and BV2 cells (B) were pretreated with the indicated concentrations of SBG (100 ug/ml and 200 ug/ml) for 30 min before incubation with LPS for 12 h. The cells were lysed and the lysates were analyzed by immunoblotting using anti cleaved caspase-3, bcl-2. Values are expressed as mean ± standard deviation (mean ± S.D.), and analyzed by one-way ANOVA and multiple comparisons were performed by Tukey HSD test. A criterion of significance was accepted and marked as *P *< 0.01 (##) and 0.001 (###). The mark of star is AC group versus NC group and the mark of pound is each treatment group versus AC group.

**Figure 7:**
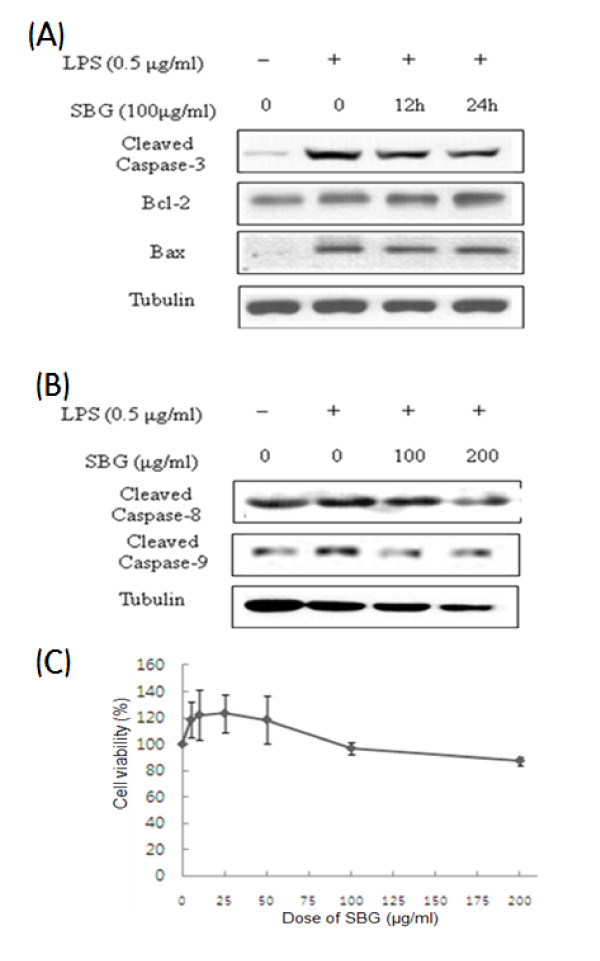
**The effect of SBG on the caspase-dependent apoptosis molecules in Raw 264.7 and BV2 cells and cell viability assay in BV2 cells**. Time dependent manner of SBG effects on the apoptosis related proteins using BV2 cells (A). SBG effects on the intrinsic apoptotic cleaved caspase 9 and extrinsic apoptosis related caspase 8 proteins in BV2 cells (B). Cell viability assay in BV2 microglia cells (C). Values are expressed as mean ± standard deviation (mean ± S.D.), and analyzed by one-way ANOVA and multiple comparisons were performed by Tukey HSD test. A criterion of significance was accepted and marked as *P *< 0.01 (##) and 0.001 (###). The mark of star is AC group versus NC group and the mark of pound is each treatment group versus AC group.

To investigate the relationship between memory impairment and apoptosis, we studied representative apoptotic molecules of mitochondrial pathways, bcl-2, bax protein and caspase 3, 8 and 9 proteins. In Raw 264.7 cells, we showed dose-dependent strong cleaved caspase-3 inhibitions and increase of bcl-2/bax protein expression ratio (Figure 6A). We also confirmed the same patterns of bcl-2 family proteins and bcl-2/bax protein expression ratio and cleaved caspase-3 in BV2 cells. Furthermore, we could see a mild increase of procaspase-3 when treated with high dose SBG (Figure 6B). Time dependent changes of cleaved caspase-3, bcl-2 and bax proteins were studied and these showed the time-dependent decrease of cleaved caspase-3, but we did not find the significant changes of bax protein and bcl-2 protein (Figure 7A). Using the results of the apoptotic changes, we proceeded with the further studies on the involvement of mitochondria. With data indicating a decrease of cleaved caspase-9 expression, no change of cleaved caspase-8, we could know that SBG has the protective effects of the deaths of microglia from apoptosis via intrinsic mitochondrial pathways (Figure 7B). And we checked the cell viabilities of SBG using BV2 cells and both low and high doses of SBG were non toxic to microglia cells (Figure 7C)

**Figure 8:**
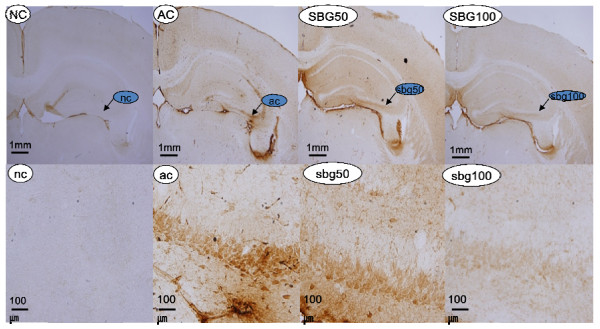
**The effect of SBG on bcl2 protein in the hippocampus cells**. NC, AC, SBG50, SBG100 (40 magnification; Bar = 100 mm), nc, ac, sbg50, sbg100 (400 magnification; Bar = 100 mm). NC, ac: Normal control group: the immunoreactive cells abundant and dark-stained; AC, ac: Aging control group: the immunoreactive cells scare and light-stained; SBG50, sbg50 and SBG100, sbg100: Group *Scutellaria baicalensis *Georgi 50 mg/kg and 100 mg/kg: the immunoreactive cells abundant and dark-stained.

For the confirmation of bcl-2 family in brain tissue, bcl-2 staining was conducted. There were numerous darkly stained immune-reactive cells in the hippocampus of the NC. The marked cells had an abundant number of primary level dendrites and long processes. Three or four level branches were visible. In the hippocampus of the mice in the AC group, the number of immune-reactive cells was less than those in the NC group. The cells were light-stained and the marked cells had few processes and lacked prominent branching. There were more immune-reactive cells in the hippocampus of the SBG treated group in contrast to the AC group and the immune-reactive cells had long processes and branches. The staining results of the SBG group were similar to that of normal control group (Figure 8).

## Discussion

Aging is a progressive process related to the accumulation of oxidative damage and the brain undergoes morphologic and functional changes resulting in the behavioral retrogression of cognition in the process of normal aging [23]. These changes are worsened by neurodegenerative diseases such as senile dementia. Senile dementia is usually accompanied by a series of abnormal alterations in neuropathology, neurophysiology and behavior. Symptoms linked with senile dementia are memory impairment, neuronal loss and plaque accumulation [24,25]. Some articles have reported that administration of D-galactose can induce oxidative damage, inflammation, caspase-mediated apoptosis and cognition impairment and abnormal biochemistry markers such as SOD, catalase and MDA in the nervous system [14,25]. Sodium nitrite (NaNO_2_) injection may cause ischemia and hypoxia in many organs in animals [15] and also induce memory-consolidating disability in mice [25]. Thus, the aging process of mice treated with D-galactose and NaNO_2 _are considerably similar to the normal aging process of humans and has served as the aging model and has been utilized to investigate the mechanism related to the aging.

In our study, after 60 days of combined administration of D-galactose and NaNO_2_, the AC group showed typical signs of senescent impairment in learning and memory function and the presence of metamorphic and necrotic neurons and induced changes in these redox-related biomarkers like a decrease in SOD, catalase activities and an increase in MDA level in the brain of mice. These alterations could have played important roles in the observed learning and memory deficits via the excess of oxidative stress. Treatment with SBG at a dose of 50 and 100 mg/kg significantly improved the cognitive impairment and decreased the generation of MDA and reduced the production of the free radical. These findings demonstrate that SBG can protect the brain against oxidative stress which was attenuated the oxidative injury induced by D-galactose plus NaNO_2_.

D-galactose combined with NaNO_2 _also induced neuronal damage in the mouse hippocampus. This would be consistent with the notion that the integrity of the central nervous system is critical for intellectual function. We also found that oral administration of SBG significantly attenuated the histological lesions that were induced by D-galactose plus NaNO_2 _in the brains of mice. This indicates that the beneficial effects of SBG on mouse cognitive deficits were primarily due to their protective effects on neurons.

Oxidative damage is one of the main factors of brain aging which is associated with neuroinflammation. Neuroinflammation had been connected to the activation of inflammatory factors iNOS and COX-2 expression. Overproduction of ROS can lead to cell death via apoptosis [26] and proteins of the bcl-2 and caspase families control the induction of apoptosis. The balance of bcl-2 family members controls survival by multiple effector mechanisms [27]. In most nonneuronal cells, anti-apoptotic bcl-2 family members act at the upstream of caspases to prevent their activation [28]. In particular, the abnormal production by glia cells of pro-inflammatory agents can disrupt nerve terminals activity and cause dysfunction and loss of synapses, which correlates with memory decline which all precede neuronal death [29].

Thus, we investigated to further identify the molecular mechanism associated with the recovery capability of the antiinflammation and antiapoptotic effects of SBG on neuronal damage with macrophage Raw 264.7 cells using western blot analysis. Expression of cyclooxygenase-2, iNOS, cleaved caspase-3, bcl-2, and bax protein were studied and NO and PGE2 production were also checked. In our study, LPS caused inflammatory response, in which iNOS, and Cox-2 levels were increased and SBG downregulated the expression of COX-2 and iNOS protein. These results suggest that SBG showed anti-inflammatory effect in the neurotoxin pathway by strongly inhibiting the Cox-2 and iNOS expression. Excessive synthesis and secretion of NO and PGE2 produced by iNOS and Cox-2 is a common feature of chronic inflammatory diseases. NO and PGE2 were also inhibited that expression by SBG treatments. These kinds of inhibition of NO and PGE2 production can be an important marker for anti-inflammatory effects. Related to the apoptotic pathways, we found that SBG also had the pro-survival effects with the strong increase of bcl-2/bax ratios and inhibition of cleaved caspase-3 expressions. For the confirmation of bcl-2 protein in brain tissue, immunohistochemistry for bcl2 protein was conducted and we found that there were more immune-reactive cells in the hippocampus of the SBG treated group in contrast to the AC group. These immunoreactive cells had long processes and branches. The staining results of the SBG group were similar to that of normal control group. In the present study, the hippocampus' expressions of bcl-2 family proteins play a pivotal role in the regulation of apoptotic cell death and inhibit production of free radicals and oxidative stress-induced neuronal death. The expression of bcl-2 family proteins were changed significantly in mice injected with D-galactose and NaNO_2_, indicating that the neuronal injury may be a result of the reduction of bcl-2 family expression in the hippocampus. Treatment with SBG (50 and 100 mg/kg) increased bcl-2 expression and bax expression in the hippocampus and then reduced oxidative stress. These effects of SBG may be the basis for its protection against pathological injury of the hippocampus and impairment of learning and memory in mice induced by D-galactose and NaNO_2_.

In conclusion, our study demonstrated that SBG administration attenuated D-galaltose and NaNO_2_-induced aging related changes in the brains of mice. SBG increased the activity of SOD and catalase and decreased the expression of iNOS and COX-2 and MDA content. SBG consequently improved the spontaneous behavior and cognitive performance. These findings about the pharmacological efficacy of SBG can contribute to brain aging research or aging-related diseases research. With these results, we can consider the possibility of SBG as a resource for new drugs or dietary supplements for patients with memory impairment, which is one of the main symptoms of several neurodegenerative diseases such as senile dementia.

## Abbreviations

SBG: *Scutellaria baicalens *Georgia; ROS: Reactive oxygen species; Cox-2: Cyclooxygenase-2; iNOS: inducible nitric oxide synthase; NO: Nitric oxide; PGE2: Prostaglandin E2; LPS: Lipopolysaccharide; NaNO_2_: Sodium nitrite; HPLC: High-performance liquid chromatography; SOD: Superoxide dismutases; CAT: Catalase; H_2_O_2_: Hydrogen peroxide; MDA: Malondialdehyde; Bcl-2: B-cell lymphoma 2.

## Competing interests

The authors declare that they have no competing interests.

## Authors' contributions

KJ, YCS and YC designed and performed the experiments. HG analyzed the data. KL, YK, SS, JY and SL drafted the manuscript. WP, YS, GK, OB, CJ, JP and YK co-designed the experiments and participated in discussion of the experimental results. SGK conceived the study, coordinated the implementation of the study. All authors read and approved the final manuscript.
